# Altered Functional Connectivity in the Motor and Prefrontal Cortex for Children With Down's Syndrome: An fNIRS Study

**DOI:** 10.3389/fnhum.2020.00006

**Published:** 2020-02-14

**Authors:** Shi-Yang Xu, Feng-Mei Lu, Meng-Yun Wang, Zhi-Shan Hu, Juan Zhang, Zhi-Yi Chen, Paulo A. S. Armada-da-Silva, Zhen Yuan

**Affiliations:** ^1^Faculty of Health Sciences, University of Macau, Macau, China; ^2^Centre for Cognitive and Brain Sciences, University of Macau, Macau, China; ^3^MOE Key Laboratory for Neuroinformation, The Clinical Hospital of Chengdu Brain Science Institute, University of Electronic Science and Technology of China, Chengdu, China; ^4^Faculty of Education, University of Macau, Macau, China; ^5^Department of Ultrasound Medicine, The Third Affiliated Hospital of Guangzhou Medical University, Guangzhou, China; ^6^Faculty of Human Kinetics, University of Lisbon, Cruz Quebrada, Portugal; ^7^Neuromechanics of Human Movement, Faculty of Human Kinetics, CIPER, University of Lisbon, Lisbon, Portugal

**Keywords:** fNIRS, cognition, Down's syndrome, brain connectivity, children

## Abstract

Children with Down's syndrome (DS) might exhibit disrupted brain functional connectivity in the motor and prefrontal cortex. To inspect the alterations in brain activation and functional connectivity for children with DS, the functional near-infrared spectroscopy (fNIRS) method was applied to examine the brain activation difference in the motor and prefrontal cortex between the DS and typically developing (TD) groups during a fine motor task. In addition, small-world analysis based on graph theory was also carried out to characterize the topological organization of functional brain networks. Interestingly, behavior data demonstrated that the DS group showed significantly long reaction time and low accuracy as compared to the TD group (*p* < *0.05*). More importantly, significantly reduced brain activations in the frontopolar area, the pre-motor, and the supplementary motor cortex (*p* < *0.05*) were identified in the DS group compared with the TD group. Meanwhile, significantly high global efficiency (*E*_*g*_) and short average path length (*L*_*p*_) were also detected for the DS group. This pilot study illustrated that the disrupted connectivity of frontopolar area, pre-motor, and supplementary motor cortex might be one of the core mechanisms associated with motor and cognitive impairments for children with DS. Therefore, the combination of the fNIRS technique with functional network analysis may pave a new avenue for improving our understanding of the neural mechanisms of DS.

## Introduction

Down's syndrome (DS) resulting from an extra 21st chromosome was first described in 1866 by John Langdon Down (Lott and Dierssen, 2010). Children who suffered from DS might exhibit disabled executive function (EF), impaired language comprehension (Martin et al., 2009), and poor learning ability and working memory (Jarrold and Baddeley, 2001). Together with cognitive dysfunctions, DS individuals also show the poor performance in motor tasks, particularly fine motor tasks involving the synchronization of hands and fingers that also require some degree of executive functioning, such as response selection/inhibition, working memory and attention (Traverso et al., 2015). EF is denoted as a set of higher-order functions that organize and regulate goal-driven behavior, which involves several brain regions including the prefrontal cortex (PFC; Diamond, 2013). Interestingly, recent studies have illustrated that the motor performance shows a significant relationship with EF in children with atypical development (Piek et al., 2004; Wassenberg et al., 2005; Hartman et al., 2010; Cao et al., 2015; Yennu et al., 2016). For example, a close relationship between the motor skill competency and EF was identified for children with DS (Horvat et al., 2013; Schott and Holfelder, 2015). In particular, the motor cortex including premotor cortex (PMC), supplementary motor area (SMA) and primary motor cortex, and the somatosensory cortex (SMC), as well as the PFC (Seitz et al., 1990; Deiber et al., 1991; Shibasaki et al., 1993; Grafton et al., 1998; Nakamura et al., 1998; Li et al., 2001; Robertson et al., 2001; O'connor et al., 2004), play an essential role in motor and executive functions (Miyachi et al., 1997; Bloedel, 2004).

The technological and methodological developments in neuroimaging such as electroencephalography (EEG), functional magnetic resonance (fMRI) and functional near-infrared spectroscopy (fNIRS) enable us to better understand the neural mechanism associated with DS during cognitive and motor tasks or at rest. Differently from EEG, both fMRI and fNIRS are based on the detection of changes in regional cerebral blood flow or blood oxygen to infer brain activity. Compared to fMRI, fNIRS has the advantages of being a portable and comfortable technique for children development studies, in which brain imaging can be performed in a quiet environment with fewer body constraints (Cheng et al., 2015; Liu et al., 2016; Scarapicchia et al., 2017).

More importantly, it has been widely recognized that cognitive tasks involve multiple spatially distributed brain regions that integrate together to generate a functional network (Fries, 2005; Bassett and Bullmore, 2006). Specifically, it was discovered that brain functional networks tend to be more random and dispersed for DS patients (Drummond et al., 2013). For example, an fMRI study has been performed, indicating that compared to TD and autistic groups, the DS participants exhibited increased synchrony between various brain networks (Anderson et al., 2013). Additional work also provided solid evidence that within-network connectivity in DS individuals is more variable in patterns as compared to that of other neurodevelopmental disorders (Vega et al., 2015). Another fMRI study illustrated that DS individuals showed increased regional connectivity in the ventral brain regions and reduced functional connectivity across the dorsal executive network (Pujol et al., 2015). Besides, Imai et al. (2014) inspected the spontaneous brain activity of sleeping DS infants to reveal the highest short-range connectivity using fNIRS. Interestingly, EEG neuroimaging was also performed associated with DS, in which Ahmadlou et al. (2013) examined whether the global organization or topology of functional brain networks was affected, suggesting that the topology of DS individuals' brain activity resembled a random rather than a small-world network. However, although previous investigations revealed dysfunctional brain connectivity in DS, most of work are carried out using resting-state recordings and the neural mechanism for disrupted brain networks during task such as fine motor tasks still remains largely unclear. In particular, further study should also be conducted to explore the topological organization of task-evoked brain networks for DS children, which can be characterized by the small-world network analysis.

In this study, fNIRS was utilized to investigate the brain cortical hemodynamic changes in children with DS during the performance of a fine motor task involving both motor and executive functions. In addition, the small-world properties of functional networks in the prefrontal and motor cortex were carefully examined to show the significant difference between the DS and TD groups. Graph theoretical network analysis has allowed us to measure the topological properties of brain networks independently of *a priori* seeds. This method models the brain as a large-scale network composed of a series of nodes (brain regions) and edges (functional connectivity between pairs of nodes; Bullmore and Sporns, 2009). Using this approach, a network could be defined as small-world networks when the graphs were with higher clustering and similar shortest path length as compared with a random network (Watts and Strogatz, 1998). We hypothesized that the DS group would demonstrate altered small-world properties compared with the TD group and expected that the DS group would be characterized by a less small-worldness with a tendency toward randomization.

## Methods

### Participants

Seven (three males, age 9.42 ± 1.61 years) DS and six (three males, age 9.00 ± 1.78 years) age- and gender-matched TD children were, respectively recruited from Guangdong Province or Macao Special Administrative Region (SAR), China to participate in this study. All children were right-handed, native Chinese speakers, and had a normal or corrected-to-normal vision. The children with DS all have their condition diagnosed by a medical doctor and confirmed by a karyotype test. All parents and caregivers were fully briefed about the study's aims and procedures and were required to give signed informed consent prior to the experiment. The study and its procedures were approved by the Ethics Committee of the University of Macau (Macao SAR, China).

### Tasks and Procedures

Before data acquisition, children were invited to the lab and become familiar with the settings and the equipment used in this study. Since participants were children, their parents or caregivers were present for the whole procedure. When performing the task, children sat on a chair in front of a 23-inch monitor with 60 Hz refresh rate being told that they were going to play a computer game called “*Catch the Hamster*.”

**Figure 1:**
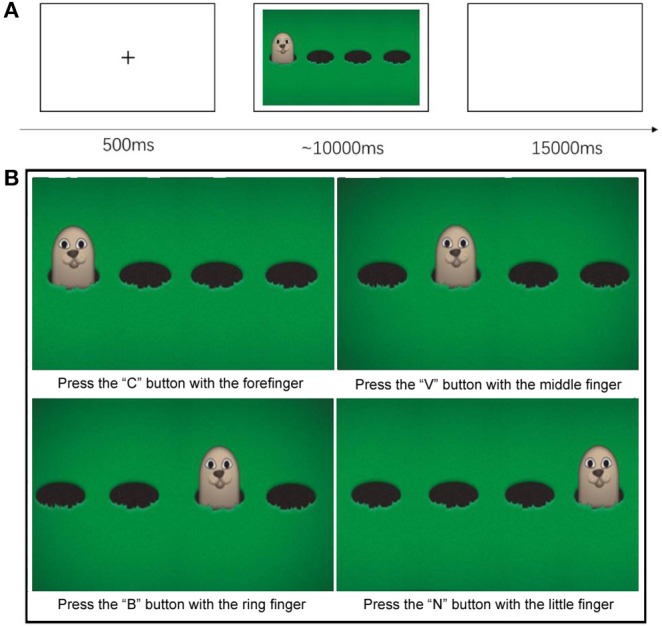
**(A)** Trials' time sequence **(B)** Children from the TD group and the DS group had to catch the Hamster by pressing the key that corresponded to its position. There will be four hamsters, and one of them will appear randomly every time. The participant need use his or her forefinger, middle finger, ring finger, and little finger of right hand to press the “C” (the first position) button, “V” (the second position) button, “B” (the third position) button, and the “N” (the fourth position) button, respectively to indicate where the hamster appeared.

During the task, participants need to press one of four keys of a computer keyboard using separate fingers of the right hand as fast as possible to indicate the correct hole where a hamster would come out. Each child was required to complete 32 trials in around 20 min. Each trial began with a warning signal consisting of a black cross showing at the center of the monitor for 500 ms. After this fixation period, a cartoon was displayed on the screen showing four hamsters' holes and a hamster coming out of one of these holes randomly every time. Children were asked to press the key matching the hole with a hamster using the index (“C key”—the first hole), middle (“V key”—the second hole), ring (“B key”—the third hole), or little (“N key”—the fourth hole) fingers of the right hand that was horizontally placed on the keyboard (Figure 1). Participants should press the corresponding key using one of four fingers as fast and accurately as possible within 10 s. Finally, a 15 s blank screen was shown between any two trials to allow hemodynamic responses to return to baseline.

The task for this experimental test was designed with the E-prime 2.0 software (Psychology Software Tools, Pittsburgh, PA), which was also used to access participants' response accuracy and reaction times automatically.

### fNIRS Recordings and Processing

#### fNIRS Systems

**Figure 2:**
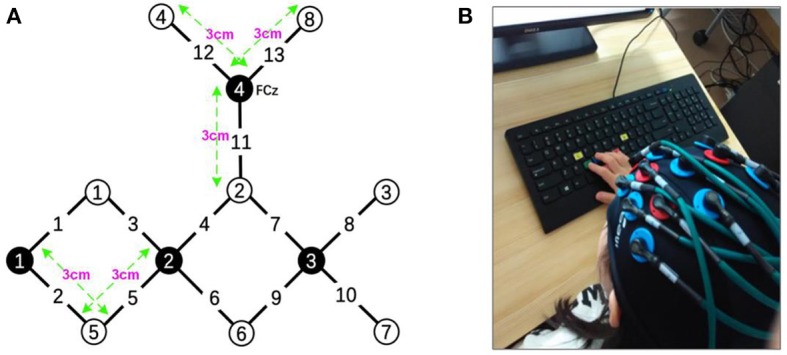
**(A)** The layout of the fNIRS system: the fNIRS head patch consisting of 4 near-infrared light source emitters (black) and 8 detectors (white). **(B)** A child was performing the task.

The experiments were performed using a continuous wave (CW) fNIRS system (CW6 fNIRS system; TechEn Inc, Milford, MA), which consisted of 4 near-infrared light source emitters and 8 detectors, as shown in Figure 2. In this system, two CW lights at wavelengths of 690 nm and 830 nm are emitted at each source optic fiber providing sensitive detection for the changes of both HbO and HbR concentrations in the human brain cortex. The distance between each source and detector pair was set to 3 cm and the sampling rate for the CW fNIRS system was 50 Hz. Notably, our CW6 system only has four laser sources and eight detectors that cannot cover the whole motor and prefrontal cortex, so we only collected hemodynamic data from the left hemisphere motor areas in this pilot study.

**Figure 3:**
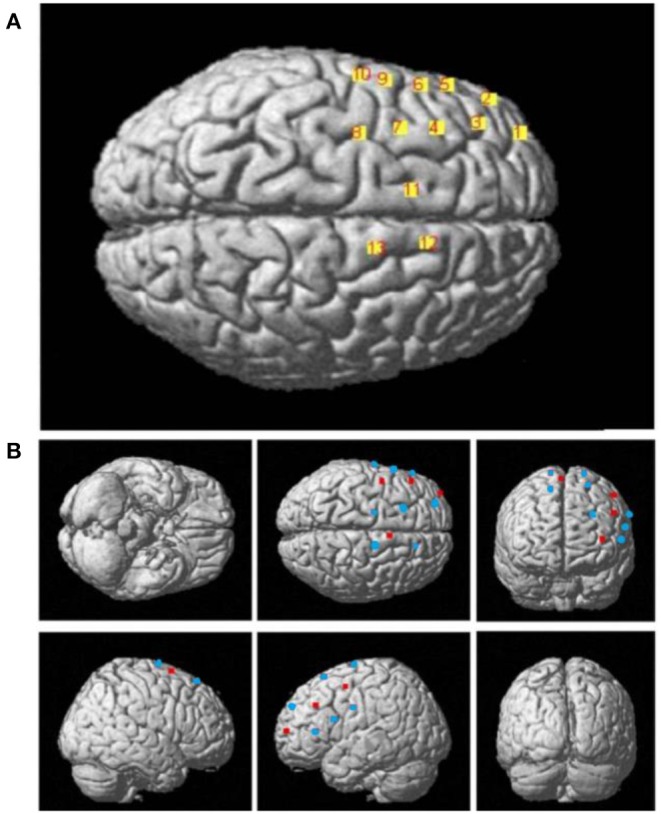
**(A)** The 3D MNI coordinates of the 13 channels. **(B)** The 3D MNI coordinates of laser sources (red) and detectors (blue).

The configuration of the four sources and eight detector pairs (Figures 2, 3) was able to generate 13 channels over the hemisphere, covering most of the left frontal cortex and motor cortex (see Table 1 for details about channels' location). The laser source four (the most anterior one) was located at Fcz according to the 10–20 standard system, serving as the reference point (Naseer and Hong, 2013). The three-dimensional (3D) positions of the optodes were measured by a 3D digitizer (PATRIOT, Polhemus, Colchester, Vermont, USA). Then the grand-averaged coordinates were processed by NIRS-SPM (Ye et al., 2009) to estimate the Montreal Neurological Institute (MNI) coordinates and associated brain regions of the optodes and channels together with the probability of the channels (Table 1). The probability is to describe how the estimated MNI coordinates are accurately corresponded to the specific brain regions processed by NIRS-SPM.

**Table 1:** Mean channel locations for the fNIRS cap.

**Channels**	**MNI coordinates (x,y,z)**	**Brodmann area**	**Probability**
CH01	−35, 59, 22	46–Dorsolateral prefrontal cortex	0.844
CH02	−50, 46, 9	45–Pars triangularis broca's area	0.545
		46–Dorsolateral prefrontal cortex	0.454
CH03	−41, 43, 35	46–Dorsolateral prefrontal cortex	0.531
		45–Pars triangularis broca's area	0.310
CH04	−38, 26, 52	9–Dorsolateral prefrontal cortex	0.827
CH05	−55, 30, 23	45–Pars triangularis broca's area	0.920
CH06	−56, 21, 29	44–Pars opercularis, part of Broca's area	0.732
CH07	−39, 12, 61	6–Pre-motor and supplementary motor cortex	0.447
		8–Includes frontal eye fields	0.286
		9–Dorsolateral prefrontal cortex	0.266
CH08	−36, −4, 66	6–Pre-motor and supplementary motor cortex	0.978
CH09	−58, 5, 39	6–Pre-motor and supplementary motor cortex	0.733
CH10	−59, −5, 44	4–Primary motor cortex	0.321
		6–Pre-motor and supplementary motor cortex	0.430
CH11	−12, 16, 70	6–Pre-motor and supplementary motor cortex	0.763
CH12	10, 23, 67	5–Pre-motor and supplementary motor cortex	0.386
		8–Includes frontal eye fields	0.613
CH13	11, 1, 75	6–Pre-motor and supplementary motor cortex	1

The fNIRS data were processed with HOMER 2 toolbox (Yang et al., 2010). To begin this process, the raw fNIRS data were converted to optical density (OD) changes (Scholkmann and Wolf, 2013). Then, OD values were corrected for motion artifacts using a spline interpolation algorithm (Scholkmann et al., 2010). The data were further band-pass filtered by a low cut-off filter frequency of 0.1 Hz and a high cut-off filter frequency of 0.01 Hz in order to minimize the physiological noise due to heart pulsation (1~1.5 Hz), respiration (0.2~0.5 Hz), and blood pressure (Mayer) waves (~0.1 Hz) as well as produce the data with the best signal-to-noise ratio. Finally, HbO concentration changes were generated using filtered OD values after normalized to zero mean and unit variance (z-scores; Zhang et al., 2010; Ding et al., 2013, 2014; Hu et al., 2018). The relative concentration changes of HbO were calculated according to the modified Beer-Lambert law (MBLL) (Lu et al., 2016) as follows:

$$\begin{array}{l} \left[\begin{array}{c} \Delta \left[HbO\right]\\ \Delta \left[HbR\right] \end{array}\right]=\frac{1}{SD}{\left[\begin{array}{c} \varepsilon_{HbO}\left(\lambda_{1}\right)DPF\left(\lambda_{1}\right)\;\varepsilon_{HbR}\left(\lambda_{1}\right)DPF\left(\lambda_{1}\right)\\ \varepsilon_{HbO}\left(\lambda_{2}\right)DPF\left(\lambda_{2}\right)\;\varepsilon_{HbR}\left(\lambda_{2}\right)DPF\left(\lambda_{2}\right) \end{array}\right]}^{-1}\left[\begin{array}{c} \Delta A\left(\lambda_{1}\right)\\ \Delta A\left(\lambda_{2}\right) \end{array}\right] \end{array}$$

where *SD* is the separation distance between source and detector, ε is the absorption coefficient, and Δ*A* is the unitless total optical density variation between a time point and at a designated baseline (time *t* = 0). The DPF at each wavelength is the unitless differential path length factor, and in this study, this value is 6.0 for the two wavelengths (690 nm and 830 nm) which was deemed as an adequate value for the present work (Scholkmann and Wolf, 2013).

In this study, only HbO signals were analyzed, since they can serve as a more sensitive indicator of changes associated with regional cerebral blood flow (Fu et al., 2014).

#### Reconstruction and Characterization of the Brain Network

The nodes and edges are two crucial components in constructing brain networks. For the present study, the nodes were denoted as the channels, whereas the edges were defined as the correlation coefficient between any two-channel pair generated by Pearson correlation analysis (Lu et al., 2017a,b). To construct the functional brain networks, the matrix of correlation coefficients was first binarized by setting a threshold value *T*, and then the correlation matrix was converted into a binary undirected graph. When the Pearson correlation coefficient is smaller than *T*, the edges can be ignored in the network. By contrast, if the correlation coefficient is equal to or larger than *T*, the two channels or nodes were connected. The thresholds were defined by the *sparsity* procedure with the GRETNA toolbox, which guarantees the same number of nodes and edges in all network matrices, allowing the assessment of relative network organization (He et al., 2009; Wang et al., 2015). After generating the binary connectivity matrix of nodes, the clustering coefficient (C_*p*_), average characteristic path length (*L*_*p*_), global efficiency (*E*_*g*_) were computed.

The clustering coefficient (C_*p*_) was defined (Watts and Strogatz, 1998),

(1) $$\begin{array}{l} C_{p}=\frac{1}{n}\sum_{i\in N}C_{i}=\frac{1}{n}\sum_{i\in N}\frac{2t_{i}}{k_{i}\left(k_{i}-1\right)} \end{array}$$

in which *C*_*i*_ is the clustering coefficient of node *i* (*C*_*i*_ = 0 for *k*_*i*_ < 2), *t*_*i*_ stands for the number of triangles around node *i*, which is a basis for measuring segregation, and *k*_*i*_ is the number of links connected to a node *i* (Rubinov and Sporns, 2010).

The average of the characteristic path length *L*_*p*_ measured the overall routing efficiency of a network (Watts and Strogatz, 1998) and was written,

(2) $$\begin{array}{l} L=\frac{1}{n}\sum_{i\in N}L_{i}=\frac{1}{n}\sum_{i\in N}\frac{\sum_{j\in N,j\neq i}d_{ij}}{n-1} \end{array}$$

in which *N* is the number of all node, *L*_*i*_ is the average distance between node *i* and all other nodes, and *d*_*ij*_ is the shortest path length between the node i and node j (Rubinov and Sporns, 2010).

The global efficiency (*E*_*g*_) measured the functional integration over all nodes in the network (Latora and Marchiori, 2001) and was denoted as

(3) $$\begin{array}{l} Eg=\frac{1}{n}\sum_{i\in N}G_{i}=\frac{1}{n}\sum_{i\in N}\frac{\sum_{j\in N,j\neq i}d_{ij}^{-1}}{n-1} \end{array}$$

in which *N* is the set of all nodes in the network and G_*i*_ is the efficiency of node i (Rubinov and Sporns, 2010).

In addition, the index σ of the small-world network was calculated as follows:

(4) $$\begin{array}{l} \sigma =\frac{C/C_{\text{rand}}}{L/L_{\text{rand}}} \end{array}$$

in which *C* and *C*_*rand*_ represent the clustering coefficients, and *L* and *L*_*rand*_ denote the characteristic path lengths of the real brain network and the comparable random network, respectively. If the value of σ was larger than 1, the network possesses the small-world characteristics (Watts and Strogatz, 1998).

### Statistical Analysis

For behavioral data, the IQ score, accuracy and reaction time were subjected to independent sample *t*-tests (two-tailed) to inspect the difference between the DS and TD groups. In particular, the children were required to perform a 32-trial task. For each trial, they need to press the button according to the position where the hamster showed up. If they press the right button, it will be counted as the right response. Otherwise, it is considered as a false response. The accuracy was determined by the number of trials with the right response divided by the whole trial number. The HbO signals and small-world properties between the two groups were also carefully examined using two-tailed paired *t*-tests.

The effect size of the *t-*test is estimated by Cohen's d and the *p*-value was corrected by the false discovery rate (FDR) (Storey, 2002). The relationship between the behavior data and the fNIRS data was generated by using Person correlation analysis. All statistical analyses were performed by SPSS 23.0 (SPSS Inc., Chicago, IL, USA) and the significance level was set to *p* < 0.05.

## Results

### Behavioral Results

**Figure 4:**
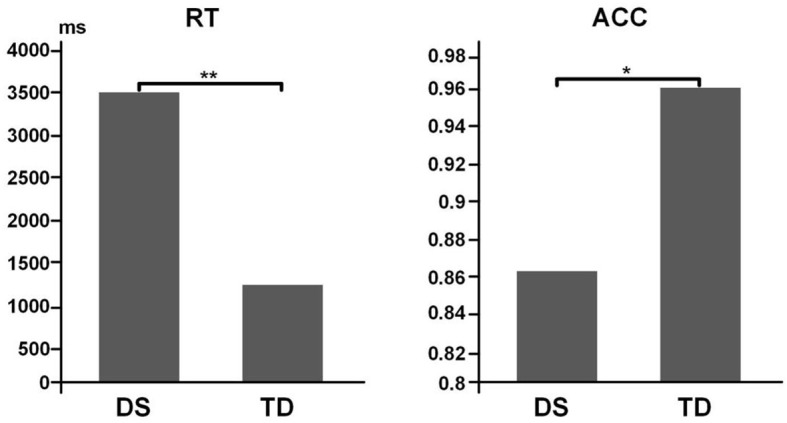
Behavior results for task performance. Left, data for RT. Right, data for ACC. **p < *0.01;* *p < *0.05*.

It was discovered that the difference in accuracy between the TD (0.958 ± 0.024) and DS groups (0.860 ± 0.030) was significant (*t* = −2.475, *p* = 0.031, *Cohen's d* = 0.14, power = 0.056). Likewise, the DS (3562.1 ± 485.7 ms) and TD (1202.4 ± 120.2 ms) groups also exhibited significant difference in the reaction time (*t* = 4.716, *p* = 0.002, *Cohen's d* = 2.53, power = 0.98; Figure 4).

### Brain Activation and Its Relationship With Behavior Performance

**Figure 5:**
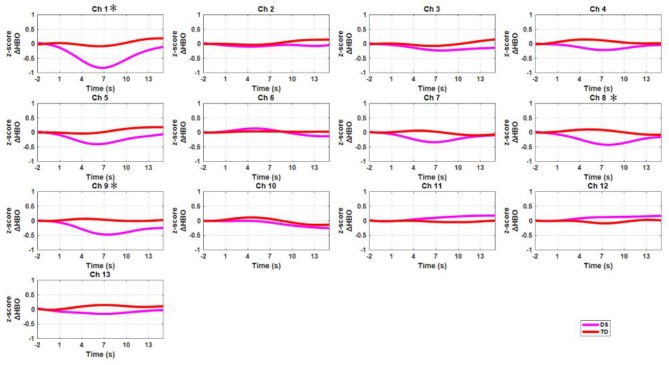
The time courses of mean HbO signals (z-scores) for all channels across all participants. The red curves represent the DS group, and the pink curves denote the TD group. The X-axis and Y-axis denotes the time (second) and HbO concentration changes (z-scores), respectively. The star means the channels that two groups showed statistical significant differences.

The grand-averaged HbO data for all channels were displayed in Figure 5 for both the DS and TD groups. After false discovery rate correction (*P*_*FDR*_ < 0.05), the two groups showed significant difference in HbO measurement in channel 1 [*t* = −3.349, *p* = 0.006, *Cohen's d* = 1.89, power = 0.87; dorsolateral prefrontal cortex (DLPFC), Brodmann area (BA)10], channel 8 (*t* = −3.204, *p* = 0.008, *Cohen's d* =1.88, power = 0.86; PMC, BA6), and channel 9 (*t* = −3.072, *p* = 0.011, *Cohen's d* = 1.74, power = 0.81; SMA, BA6).

**Figure 6:**
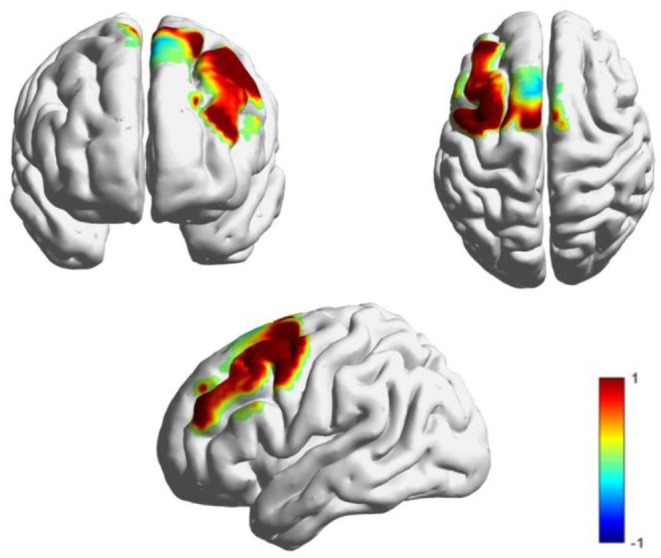
T-maps for the cortical activation difference between the TD and DS groups.

As shown in Figure 6, the *t*-values of the HbO signal difference between the DS and TD groups were visualized on a brain cortex template by using the Xjview toolbox (http://www.alivelearn.net/xjview) and BrainNet Viewer toolbox (Xia et al., 2013).

**Figure 7:**
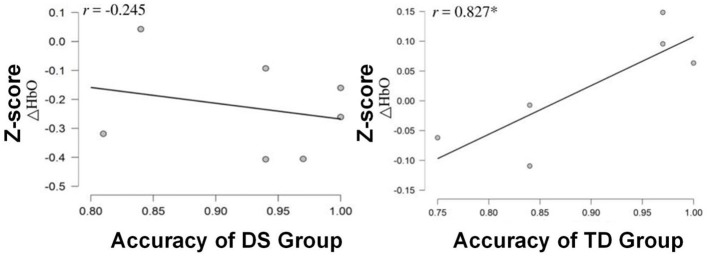
Scatterplots and correlation coefficient (r) between the z-scores of HbO changes for channel 8 (the PMC) and ACC for each group of the children. *p < *0.05*.

To inspect the relationship between task performance and HbO data, the Pearson's correlation analysis was performed. It was discovered that only the TD group showed a significant positive correlation between the HbO measurement in channel 8 and task accuracy (*r* = 0.827, *p* < 0.05; Figure 7). However, it was not the case for the DS group, in which no significant correlation was identified.

### Brain Network Analysis

**Figure 8:**
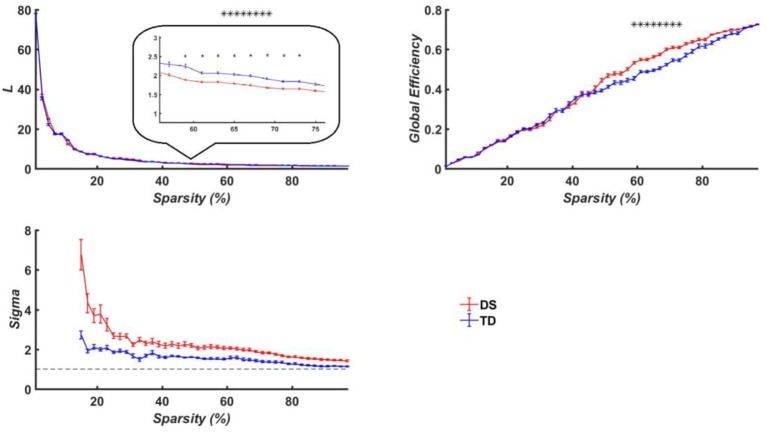
Brain network properties of the TD the DS groups. Top left is for averaged path length (*L*_*p*_); top right is the averaged node degree; bottom left is the measure of small-worldness, and the dashed curve is 1. The curves show the network indicators under different thresholds, in which the red represents the DS group while the blue denote the TD group. The horizontal axis denotes the threshold values while the vertical axis denotes the network properties indicators. Here the asterisks denote *p* < 0.05.

The average path length (*L*_*p*_), global efficiency (*E*_*g*_) and the measure of small-worldness (σ) were, respectively calculated for both the DS and TD groups, which were provided in Figure 8 as a function of the sparsity (i.e., threshold *T*). Since the σ of constructed brain functional networks for both groups was larger than 1 as compared to that of matched random networks (Figure 8), the functional brain networks for the two groups exhibited small-world property. More importantly, we discovered that the DS group manifested significantly larger *E*_*g*_ and shorter *L*_*p*_ than the TD group when the threshold *T* was ranged between 0.59 and 0.73.

## Discussion

In this study, the small-world analysis combined with fNIRS recordings was conducted to inspect the differences in brain activation and networks along the motor and prefrontal cortex between the TD and DS children, which involves both the motor and cognitive functions, particularly the EF.

We discovered that the TD group exhibited significantly higher brain activation in the DLPFC and PMC (channels 1, 8, and 9) as compared to the DS group during a fine motor task (Figure 5). Interestingly, the DLPFC and the PMC are related to motor and executive functions (Haber, 2003; Leh et al., 2010; Stein et al., 2017). Previous studies employing similar fine motor tasks have also demonstrated that the DLPFC plays an essential role in performing these type of tasks that require EF in order to correctly plan the more complex motor actions (Robertson, 2007; Yogev-Seligmann et al., 2008; Sturm et al., 2016; Sachs et al., 2017).

In addition, no significant relationship was identified between fNIRS data and task performance in DS children. However, a strong positive correlation was revealed between the level of activation in channel 8 and task accuracy in TD children. Our new findings illustrated that the premotor cortex is vital for the performance of the task employed in this study. The impaired ability to activate this cortical brain region might cause lower task performance in DS children.

Further, the motor and executive functions deficits in the DS group were demonstrated by altered brain functional connectivity. In particular, the functional brain networks of the DS group exhibited higher global efficiency and shorter path length as compared to those of the TD group, which demonstrated the tendency toward a more random connectivity. Consequently, the brain networks with higher *E*_*g*_ and shorter *L*_*p*_ might result in worse performance in the motor task for the DS group.

Besides the small sample size, only the DLPFC and the PMC was inspected in this study, thus limiting our ability to ascertain the impact from other brain regions. While this is a limitation of fNIRS neuroimaging technology, it will allow us to conduct a task-based study in DS children. This study offers some insight into the mechanism of the development of the fine motor function with the children with DS from the perspective of brain networks.

## Data Availability Statement

The datasets generated for this study are available on request to the corresponding author.

## Ethics Statement

The studies involving human participants were reviewed and approved by Ethics Committee of the University of Macau (Macao SAR, China). Written informed consent to participate in this study was provided by the participants' legal guardian/next of kin.

## Author Contributions

ZY, F-ML, and S-YX designed research and wrote the paper. Z-SH and M-YW performed research and analyzed data. PA-S reviewed and discussed the data, proofread the manuscript, and co-wrote its final version. JZ and Z-YC provided the invaluable suggestion and revised this manuscript.

### Conflict of Interest

The authors declare that the research was conducted in the absence of any commercial or financial relationships that could be construed as a potential conflict of interest.
